# Transverse Cervical Vessels: A Viable Option for Microsurgical Reconstructions in Vessel‐Depleted Necks

**DOI:** 10.1002/ccr3.73811

**Published:** 2026-10-30

**Authors:** Julio Camilo Torres Gonzalez, María Camila Rojas Gómez, Andrés Alberto Álvarez Tamayo, Rogers Leonardo Baquero, Martha Luz Torres Pabón, Juan Carlos Olaya Osorio, Gonzalo Mallarino Restrepo

**Affiliations:** ^1^ Hospital Universitario Mayor–Méderi Bogotá Colombia; ^2^ Head & Neck Surgery Department Hospital Universitario Mayor–Méderi Bogotá Colombia; ^3^ Plastic Surgery Department Hospital Universitario Mayor–Méderi Bogotá Colombia

**Keywords:** free flap, head and neck reconstruction, microvascular reconstruction, reconstructive surgery, transverse cervical vessel, vessel‐depleted neck

## Abstract

Transverse cervical vessels are a safe and reliable recipient alternative for microvascular reconstruction in vessel‐depleted necks. In three complex head and neck cases, all anastomoses succeeded, with 100% flap survival, no re‐exploration, and no airway or swallowing sequelae at a minimum 2‐month follow‐up.

## Graphical abstract

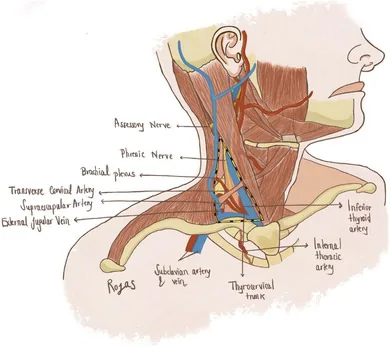

## 1 Introduction

The incidence of head and neck cancer has been increasing, currently ranking as the sixth most common malignancy worldwide. Approximately 900,000 new cases are diagnosed each year, and the disease carries a mortality rate close to 50%. More than 90% of head and neck cancers are squamous cell carcinomas, which frequently exhibit aggressive behavior [1, 2, 3]. In the vast majority of cases, management requires extensive surgical resection followed by reconstruction with a free flap. In some instances, surgical treatment is complemented with radiotherapy and/or chemotherapy as part of a multimodal therapeutic approach aimed at achieving the best possible oncologic resection while preserving as much healthy tissue as possible [2, 4].

Despite the rich vascularization of the head and neck region, patients with a history of surgery in this area may have sustained damage or alteration to branches of the external carotid artery, which are commonly used as recipient vessels. Likewise, prior adjuvant radiotherapy may induce extensive fibrosis of tissues and vessels, making any subsequent dissection extremely difficult or even impossible [4, 5].

The surgical challenge increases when, despite multimodal treatment, the patient develops tumor recurrence, second primary tumors, or postoperative complications, as a second free flap may be required. In such situations, reconstructive plastic surgeons face an additional obstacle: a scarred and fibrotic neck, commonly referred to as a “frozen neck” or “vessel‐depleted neck”, which significantly limits the availability of recipient vessels for successful secondary microvascular reconstruction [4, 5, 6].

In this context, plastic surgeons have been compelled to explore alternative recipient vessel options that may enable head and neck reconstructions in these complex scenarios. The objective of this article is to present the clinical outcomes of a series of patients with vessel‐depleted necks reconstructed with free flaps using anastomoses to the transverse cervical vessels, demonstrating that this is a viable additional option to consider for such reconstructions.

## 2 Case History/Examination

### 2.1 Case 1

A 79‐year‐old male patient was referred for a large cell carcinoma arising from the left parotid gland, with extension to the preauricular region and measuring approximately 10 × 4 cm. In coordination with the head and neck surgery team, a parotidectomy with extensive oncologic resection was performed, including lymph node dissection of Levels III and V, partial auriculectomy and left submandibular gland excision, along with ligation of the external carotid artery and external jugular vein due to marginal tumor involvement.

### 2.2 Case 2

A 74 year old female patient with a history of total thyroidectomy and cervical lymph node dissection for papillary carcinoma, followed by adjuvant radiotherapy 10 years earlier and a right radical neck dissection 6 years later due to tumor recurrence presented again with a new relapse. CT scan imaging revealed a solid mass measuring 27 × 23 mm with locoregional involvement, airway compromise, and compression of the internal jugular vein. Due to laryngeal and esophageal involvement, she underwent total pharyngolaryngectomy with left radical neck dissection. Surgical dissection was technically challenging because of extensive fibrosis secondary to prior treatments, and no viable recipient vessels were identified for the upcoming reconstruction.

### 2.3 Case 3

A 47‐year‐old female patient with a history of head and neck carcinoma treated with chemoradiotherapy underwent total glossectomy, mandibulotomy and bilateral neck dissection for squamous cell carcinoma of the tongue, with floor‐of‐mouth reconstruction using an anterolateral thigh (ALT) free flap anastomosed to the superior thyroid artery and external jugular vein, followed by adjuvant radiotherapy. She subsequently developed osteoradionecrosis and osteomyelitis of the mandibular symphysis with an orocutaneous fistula, requiring multiple washouts, debridements and intravenous antibiotic therapy for a polymicrobial infection. Histopathological analysis confirmed osteoradionecrosis with osteomyelitis, with no evidence of tumor recurrence. Approximately 1 year after the initial reconstruction, following multidisciplinary board review, segmental resection of the necrotic symphysis and mandibular reconstruction with a reconstruction plate were performed, and soft‐tissue coverage was achieved in the same procedure with a second ALT free flap. Because the conventional recipient vessels had been used in the previous reconstruction and the neck had been irradiated, the flap was anastomosed to the transverse cervical vessels. See Table 1 for clinical case details.

**TABLE 1:** Clinical cases characteristics.

Characteristics	Case 1	Case 2	Case 3
Age	79 year old	74 year old	47 year old
Sex	Male	Female	Female
Diagnosis	Large cell carcinoma of the left parotid gland with preauricular extension	Papillary thyroid carcinoma	Osteoradionecrosis and osteomyelitis of symphysis
Oncologic history	No previous oncologic history	Total thyroidectomy with cervical lymph node dissection followed by adjuvant radiotherapy; 6 years later underwent a right radical neck dissection for tumor recurrence	Prior head and neck carcinoma treated with chemoradiotherapy. Squamous cell carcinoma of the tongue managed with total glossectomy, mandibulotomy, bilateral neck dissection and ALT flap reconstruction (superior thyroid artery/external jugular vein), followed by adjuvant radiotherapy
Resection performed	Extensive resection + ND III–V, partial auriculectomy + SMG excision + ECA/EJV ligation	Total pharyngolaryngectomy + left radical neck dissection	Segmental resection of necrotic mandibular symphysis and reconstruction plate (after multiple washouts, debridements and IV antibiotics)
Reconstructive technique	ALT flap	ALT flap	ALT flap
Recipient vessels	Transverse cervical vessels	Transverse cervical vessels	Transverse cervical vessels
Type of microanastomosis	End‐to‐end, 8–0 Ethilon	End‐to‐end, 8–0 Ethilon	End‐to‐end, 8–0 Ethilon
Complications	No complications	Small circumferential dehiscence healed by secondary intention (Clavien‐Dindo I)	No complications
Outcome	Complete flap survival. No complications. Preserved airway and swallowing function	Complete flap survival; Preserved airway and swallowing function	Complete flap survival; no complications. Preserved airway and swallowing function
Duration of Follow‐Up	2 Months	2 Months	12 Months

Abbreviations: ALT flap, anterolateral thigh flap; ECA, external carotid artery; EJV, external jugular vein; ND, neck dissection; SMG, submandibular gland.

## 3 Methods (Differential Diagnosis, Investigations and Treatment)

All cases underwent multidisciplinary clinical evaluation and physical examination. Additional studies were performed according to each case to determine the extent of disease and guide surgical planning: contrast‐enhanced computed tomography (CT) in Case 2 and histopathological analysis in Cases 1 and 3, as detailed in the case descriptions. Prior surgeries, radiotherapy and/or extensive oncologic resection resulted in challenging cervical anatomy in all cases, with a vessel‐depleted (“frozen”) neck that limited the availability of conventional recipient vessels for free flap reconstruction.

Based on recipient vessel availability, the transverse cervical artery and vein were selected in all three cases for reconstruction using an anterolateral thigh (ALT) free flap. The ALT flap was chosen because it provided an adequately sized skin paddle for defect coverage and sufficient pedicle length to reach these alternative recipient vessels. The surgical team's established experience with this flap further supported its selection in these complex scenarios. End‐to‐end microvascular anastomoses were performed using 8–0 nylon sutures under an operating microscope. Postoperative management included standardized flap monitoring and institutional antibiotic and antithrombotic protocols, with a minimum clinical follow‐up of 2 months, focused on flap viability and surgical complication assessment using the Clavien‐Dindo classification in cases where complications were present. Airway and swallowing function were assessed in all cases at each follow‐up visit by clinical examination. See Table 2 for a review of alternative recipient vessels in head and neck reconstruction.

**TABLE 2:** Alternative recipient vessels in head and neck reconstruction.

Recipient vessel option	Indication^a^	Advantages	Disadvantages
Contralateral carotid system	Defects of the mid and lower thirds of the face when the ipsilateral system is not viable [7]	Close proximity to recipient arteries and easy access to the internal jugular vein and its branches [7] The internal jugular vein has a high success rate due to its high‐volume blood flow, which can clear small thrombi [7] The internal jugular vein can support more than two end‐to‐side anastomoses [7] The superior thyroid artery has an accessible position and is easy to dissect [7]	Small diameter of the superior thyroid artery (~1.5 mm) [7] The external jugular vein has low flow and a small caliber [7] The external jugular vein is more prone to compression [7] The carotid artery is more difficult to dissect because it is not exposed during neck dissection, and clamp time is limited [7]
Superficial temporal vessels	Intraoral, upper‐ and mid‐face, and scalp defects [8]	Generally unaffected by prior surgeries or radiation due to their location [7] Possibility of using retrograde flow in reoperations [8]	The superficial temporal vein is thin and fragile [8] The superficial temporal artery is tortuous and prone to vasospasm [8] A subcutaneous tunnel is often required to avoid pedicle compression [8] Pedicle length is limited for hypopharyngeal or laryngeal defects [8]
Internal mammary vessels	Defects of the lower third of the face and neck when more superficial vessels are unavailable [9]	Reliable vascular supply [9] Greater anatomical consistency compared to the thoracoacromial system [9] Artery and vein diameters appropriate for recipient vessels [9]	Risk of entering the pleural space during dissection [9] Small venous caliber, especially below the third rib, which complicates microvascular anastomosis [9] Incompatible with simultaneous use of the ipsilateral pectoralis major flap [9]
Thoracoacromial system	Defects of the upper, middle, and lower thirds of the face when more superficial vessels are unavailable [10]	Adequate caliber for anastomosis at the clavicular level [10] Allows transposition over the clavicle to reach the ipsilateral neck, avoiding the need for venous grafts [10]	High venous variability that complicates dissection [10] Possible loss of the main vascular pedicle of the pectoralis major, limiting future use of this flap [10] Anatomical distortion of the thoracoacromial system if the pectoralis major has been previously elevated [10]
Transverse cervical vessels	Defects of the lower third of the face and neck when more superficial vessels are unavailable [11]	Generally remain intact after prior surgeries or radiotherapy [6] Longitudinal alignment that facilitates anastomosis [6] Diameter > 2 mm and adequate flow, similar to branches of the external carotid artery [6] Less affected by atherosclerosis [6] Location in the lower neck improves access and microscope positioning [7]	Higher risk of arterial rupture due to the technical difficulty of dissecting it independently from the neck [7] Requires removal of surrounding scar tissue, increasing the risk of intimal injury and creating a dead space at the root of the neck, which may become a potential site for infection [7]

^a^ It is essential to consider individual patient factors, such as surgical history, vessel availability, and characteristics of the defect to be reconstructed—when selecting the most appropriate recipient vessel.

## 4 Outcome and Follow‐Up

In all three cases, microvascular reconstruction was successfully performed using the transverse cervical vessels as recipient vessels. Adequate intraoperative blood flow and flap perfusion were confirmed in all cases, and no intraoperative complications related to vessel dissection or microvascular anastomosis occurred. Intraoperative findings demonstrated appropriate vessel caliber, length, and positioning for anastomosis (Figure 1).

**FIGURE 1:**
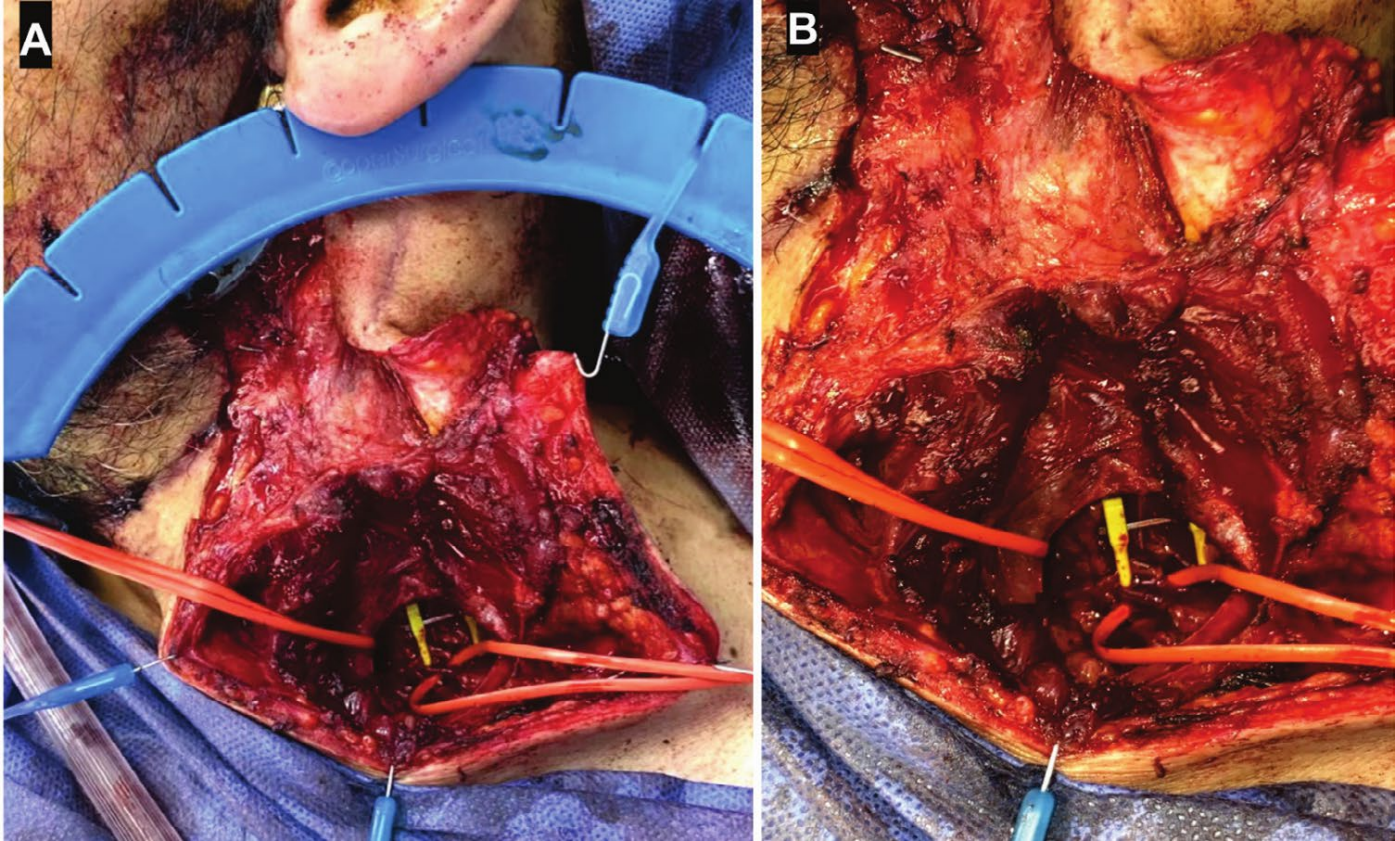
Case 1. (A) Intraoperative view of the left neck following oncologic resection, showing an extensive cervical dissection field. The transverse cervical artery and vein are identified in the inferior aspect of the wound, situated deep and posterior to the sternocleidomastoid remnant. (B) Magnified view illustrating the course and separation of the transverse cervical vessels. Vessel loops delineate the arterial and venous branches, allowing clear visualization of adequate vessel length and caliber suitable for microvascular anastomosis.

Postoperatively, all flaps were monitored for signs of vascular compromise by clinical assessment of color, capillary refill, temperature, and turgor. Flap viability was maintained throughout follow‐up in all cases. No episodes of partial flap compromise, venous congestion, arterial or venous thrombosis, or flap loss were observed, and no cases required re‐exploration or reintervention. Donor sites were evaluated at each follow‐up visit, and no donor‐site complications such as hematoma, seroma, infection, or dehiscence occurred.

The only postoperative event recorded was a minor wound dehiscence along the flap margin in Case 2. There were no signs of flap ischemia, and the dehiscence was managed conservatively and healed by secondary intention. Airway and swallowing function were assessed in all cases during follow‐up, and no functional sequelae attributable to the choice of recipient vessels were identified. During follow‐up (2–12 months), flap survival was 100%.

## 5 Discussion

The multiple challenges involved in reconstructing a vessel‐depleted neck, whether due to extensive oncologic resection, multiple prior surgeries, and/or previous radiotherapy, are present in approximately 7% of head and neck reconstruction cases [6]. In these complex scenarios, the appropriate selection of recipient vessels is essential to ensure flap survival and minimize complications [12].

Traditionally, branches of the external carotid artery and the superficial temporal artery have been the most frequently used options. However, in previously operated or irradiated necks, the vessels present several limitations, such as variability in caliber and length, increased susceptibility to atherosclerosis, and technical difficulties related to fibrosis and distortion of the anatomical planes [5, 6, 13, 14]. These conditions may compromise both dissection and the quality of the anastomosis.

In contrast, the transverse cervical vessels represent a highly reliable option that, in many situations, may surpass traditionally selected vessels in the context of a vessel‐depleted neck. They often remain intact even after prior cervical surgeries or radiotherapy, as the posterior triangle is rarely included in oncologic resections or radiation fields [6, 11]. Their longitudinal course facilitates anastomosis and they consistently demonstrate a diameter greater than 2 mm and an adequate blood flow, comparable to that of external carotid branches, thereby ensuring excellent anastomotic quality [6]. They are also less affected by atherosclerosis, reducing the risk of thrombosis [6], and their location in the lower neck offers improved surgical access and microsurgical positioning, making the technique more comfortable and safer [7, 8, 9]. The anatomical location and relationships of the transverse cervical vessels within the posterior cervical triangle, which contribute to their relative preservation and surgical accessibility in these scenarios, are illustrated in Figure 2.

**FIGURE 2:**
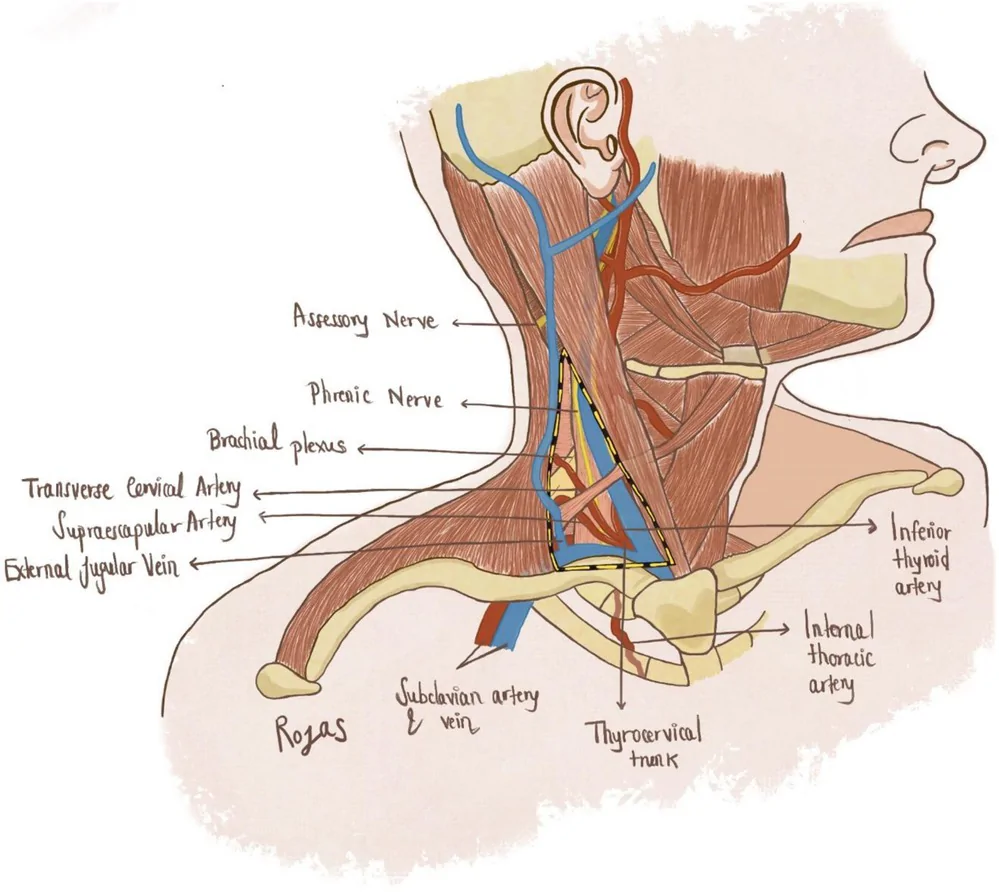
Transverse cervical vessels. Anatomy of the transverse cervical vessels and their relationship with the neurovascular structures of the posterior cervical triangle, demonstrating their accessibility and usefulness as recipient vessels in microsurgical reconstruction of frozen necks. Illustration created by María Camila Rojas Gómez, adapted and reproduced with permission from Sisti et al. [15]. © 2020 Cleveland Clinic Center for Medical Art & Photography.

Our cases consistently illustrate these advantages. In all three cases, the transverse cervical vessels remained available despite prior surgeries, radiotherapy and/or extensive oncologic resection. Even in Case 1, which involved an extensive defect secondary to parotidectomy, the posterior cervical triangle remained preserved, allowing straightforward identification and use of these vessels as recipients. Adequate flow, ease of dissection, and the absence of significant fibrosis along their course contributed to safe anastomosis in all cases [7, 10].

Although their dissection is generally straightforward due to their low anatomical variability, it must be performed with caution given their proximity to structures such as the thyrocervical trunk, the phrenic nerve, the spinal accessory nerve, the brachial plexus, and the thoracic duct, particularly for surgeons who are still within their learning curve [14]. Despite these considerations, studies such as that by Schettini et al. have demonstrated lower complication rates when compared with carotid vessels [5].

In all three cases, microvascular reconstruction was successful, with 100% flap survival and no airway or swallowing sequelae. No complications related to the dissection or to the anastomosis of the transverse cervical vessels were observed, and none of the cases has required reintervention during follow‐up. Only one case presented a minor complication, a wound dehiscence, which was managed conservatively with local care and secondary intention closure. These findings support the use of transverse cervical vessels as a viable alternative for reconstruction of vessel‐depleted necks. Limitations of this case series are its small descriptive design (three cases, single flap type), the short follow‐up period, the lack of a comparison group, the absence of formal assessment of speech, oral competence and quality of life using validated instruments, and the absence of observed flap failures, which together restrict the generalizability and risk assessment of our findings. Larger cohorts with longer follow‐up are needed to confirm these results.

Transverse cervical vessels are a safe, reliable, and effective option for microvascular reconstruction in patients with vessel‐depleted necks, particularly in those with multiple prior surgeries or radiotherapy. Although their dissection requires caution due to their proximity to critical structures, appropriate preoperative planning and meticulous technique help minimize these risks. The available evidence, together with our experience, supports their use as a robust alternative and they should be considered early in the reconstructive plan in complex neck reconstruction when traditional vessels are unavailable [7, 8, 9, 10, 14, 16].

## Author Contributions

**Julio Camilo Torres Gonzalez:** conceptualization, data curation, formal analysis, investigation, writing – original draft, writing – review and editing. **María Camila Rojas Gómez:** conceptualization, data curation, formal analysis, investigation, methodology, writing – original draft, writing – review and editing. **Andrés Alberto Álvarez Tamayo:** conceptualization, data curation, formal analysis, investigation. **Rogers Leonardo Baquero:** conceptualization, data curation, formal analysis, investigation. **Martha Luz Torres Pabón:** conceptualization, data curation, formal analysis, investigation. **Juan Carlos Olaya Osorio:** conceptualization, data curation, formal analysis, investigation. **Gonzalo Mallarino Restrepo:** conceptualization, data curation, formal analysis, investigation, methodology.

## Funding

The authors have nothing to report.

## Disclosure

Permission to reproduce material: One figure was adapted and reproduced with permission from Sisti et.al. [15]. 2020 Cleveland Clinic Center for Medical Art & Photography.

## Ethics Statement

This study was approved by the Institutional Ethics Committee of Hospital Universitario Mayor Mederi, Bogotá, Colombia (approval code CEISH‐2024009).

## Consent

Written informed consent was obtained from all patients for publication of clinical information and images.

## Conflicts of Interest

The authors declare no conflicts of interest.

## Data Availability

The data supporting the findings of this study are available from the corresponding author upon reasonable request. Due to the nature of this study, involving identifiable patient information, the data are not publicly available.
